# Mini Review: Opposing Pathologies in Cancer and Alzheimer's Disease: Does the PI3K/Akt Pathway Provide Clues?

**DOI:** 10.3389/fendo.2020.00403

**Published:** 2020-06-23

**Authors:** Rachel M. Barker, Jeff M. P. Holly, Kalina M. Biernacka, Shelley J. Allen-Birt, Claire M. Perks

**Affiliations:** ^1^IGFs & Metabolic Endocrinology Group, Bristol Medical School, Translational Health Sciences, Southmead Hospital, University of Bristol, Bristol, United Kingdom; ^2^Molecular Neurobiology Group, Bristol Medical School, Translational Health Sciences, Southmead Hospital, University of Bristol, Bristol, United Kingdom

**Keywords:** cancer, Alzheimer's, PI3K/Akt pathway, IGF-1, insulin, LRP1, PIN1, p53

## Abstract

This minireview is a brief overview examining the roles of insulin-like growth factors (IGFs) and the PI3K/Akt pathway in two apparently unconnected diseases: Alzheimer's dementia and cancer. For both, increased age is a major risk factor, and, in accord with the global rise in average life expectancy, their prevalence is also increasing. Cancer, however, involves excessive cell proliferation and metastasis, whereas Alzheimer's disease (AD) involves cell death and tissue destruction. The apparent “inverse” nature of these disease states is examined here, but also some important commonalities in terms of the PI3K/Akt pathway, glucose utilization and cell deregulation/death. The focus here is on four key molecules associated with this pathway; notably, the insulin receptor substrate 1 (IRS-1), cellular tumor antigen p53 (p53), peptidyl-prolyl cis-trans isomerase NIMA-interacting 1 (PIN1) and low-density lipoprotein receptor–related protein-1 (LRP1), all previously identified as potential therapeutic targets for both diseases. The insulin-resistant state, commonly reported in AD brain, results in neuronal glucose deprivation, due to a dampening down of the PI3K/Akt pathway, including overactivity of the mammalian target of rapamycin 1 (mTORC1) complex, hyperphosphorylation of p53 and neuronal death. This contrasts with cancer, where there is overstimulation of the PI3K/Akt pathway and the suppression of mTORC1 and p53, enabling abundant energy and unrestrained cell proliferation. Although these disease states appear to be diametrically opposed, the same key molecules are controlling pathology and, with differential targeting of therapeutics, may yet provide a beneficial outcome for both.

## Background

In 2018 there were 17 million new cases of cancer and 9.6 million deaths worldwide (1). One of its most common forms is breast cancer, a leading cause of cancer mortality worldwide (2), with over two million new cases in 2018. Dementia is also a major cause of suffering and death globally, with 9.9 million new cases estimated each year (3); 60–70% of these are diagnosed as Alzheimer's disease (AD) (4). AD and breast cancer, as examples of each disease spectrum, are contrasted here with respect to differences in the PI3K/Akt pathway. By comparing four specific key molecules, we hope to provide some insight into potential, differential therapeutic targeting. Although, due to the limitations of a mini-review we needed to narrow our selection, we acknowledge that additional molecules contributing to the inverse nature of these pathologies have also been reviewed previously (5).

Every normal cell in the body will acquire mutations over a lifetime, which may result in cancer. It has been clear for many years that the initiating mutations and neoplastic transformation may occur decades before symptoms become present and the cancer is diagnosed. Most breast cancers are epithelial tumors that develop from cells lining ducts or lobules: carcinoma *in situ*, and are located exclusively in the breast, tending to be detected by routine physical examination or mammography. Invasive breast cancer can spread however, to most organs, with the main sites being the lungs, liver, bone and brain. There are five main subtypes of breast cancer, depending on the expression of the estrogen, progesterone and human epidermal growth factor receptor 2 (HER2) receptors which dictate treatment strategies (6). One mutational profile often observed in many cancers is hyperactivity of the PI3K/Akt signaling pathway leading to deregulated control of cell proliferation (7). Another common feature associated with cancer risk and progression is chronic inflammation, which can be initiated by triggers, such as infections, obesity and autoimmune diseases, the effects of which can be mediated by cytokines, such as tissue necrosis factor (TNF) and interleukins (IL-1 and 6) (8).

As for cancer, the diagnosis of AD usually occurs long after the onset of neuropathology, often 10–20 years later, mainly because symptoms do not generally become evident until the brain has been severely compromised. Loss of short-term memory is usually the first symptom; later, cognitive failure and confusion, and finally an inability to carry out tasks required for successful daily living. Its two defining brain pathologies are the presence of amyloid plaques, comprised mainly of the toxic peptide Aβ42 (processed from the amyloid precursor protein (APP), which quickly fibrillises and deposits in the parenchyma of the brain, and hyperphosphorylated tau, which accumulates within neurones into neurofibrillary tangles (NFT). The parallel spread of these two pathologies across the brain, occurs over a long period before clinical symptoms become evident. Until recently, this has made early diagnosis and assessment of treatment effectiveness difficult. Positron emission tomography (PET) scans with ligands which register amyloid and NFT, as well as markers of neuroinflammation, are now available, helping diagnosis, clinical trial investigation and basic scientific discovery (9). Recent investigations with PET ligands in living patients suggest that symptoms are noticeable when amyloid and NFT both reach sufficiently high levels (10). The brain, separated from the peripheral immune system by the blood-brain-barrier (BBB), relies on its innate immune system for defense, this includes production of Aβ42 peptide (11) and activation of the resident macrophages, microglia, resulting in neuroinflammation, neuronal loss and ultimately death (12). Unless constantly cleared, Aβ42 forms plaques, whilst toxic, soluble oligomeric forms also contribute to neuronal death. Familial forms of AD with mutations with increased Aβ42 formation, led to the “amyloid cascade hypothesis” (13) where amyloid precipitates the full spectrum of pathology and symptoms. Although clearly still very useful, this is undergoing re-appraisal in terms of the non-familial or common sporadic form (14, 15).

Whilst most cancers, including breast cancer, involve apparently unrestrained cell proliferation, AD involves cell loss. Neurones in the brain, are terminally differentiated post-mitotic cells, which if forced into cycle re-entry usually die (16). Cancer is associated with an increased glucose uptake by tumor cells, that is preferentially converted to lactate fermentation: a phenomenon known as the Warburg effect (17). The Warburg effect co-ordinates a number of cellular processes however, in addition to lactate fermentation, including preventing damage from reactive oxygen species (ROS), ensuring that cancer cells have a supportive microenvironment for cell proliferation (18). By contrast, AD is associated with an early reduction of glucose uptake and utilization in certain areas of the brain (19, 20). Due to its commonly seen insulin-resistance brain profile, AD is sometimes referred to as Type3 diabetes mellitus (T3DM) (19–22).

Despite the apparently different pathologies, we investigate here aspects of insulin/IGF signaling and the PI3K/Akt pathway that may determine these differences and briefly explore underlying commonalities between the mechanisms which play a role in the two disease states. Glucose intolerance increases generally with age (16, 17) and this is thought to be due to insulin-resistance, commonly observed in older adults (18, 19). Despite the opposing pathologies, cancer and AD have common risk factors such as aging, diabetes, obesity, smoking (23) and lack of exercise, each of which is also associated with insulin-resistance (24–27). Yet, as noted, although the AD brain often develops insulin-resistance, tumor cells generally do not. Here, we discuss normal cellular energy homeostasis and how this differs in cancer and AD.

## Regulation and Function of Insulin and IGF-1 in Health, Cancer and AD

The main source of insulin is that secreted from the beta-cells of the pancreas in response to food; this normalizes the levels of blood glucose, by inducing its target tissues, liver, muscle, and fat cells to increase glucose uptake. IGF-I is secreted by the liver in response to growth hormone, and its circulating levels remain constant via its unique interaction with its IGF binding proteins (IGFBPs) (28). Unlike insulin, IGF-I (and IGF-II) are also made in most cells of the body, where they play key roles in growth, survival and metabolism. During an insulin-resistant state the usual normalizing processes are inhibited, leading to increased levels of circulating insulin and glucose. This also leads to a stimulation of hepatic IGF-I synthesis (29), and downregulation of IGFBPs-1 and−2, resulting in an increased bioavailability of IGF (30).

**Figure 1:**
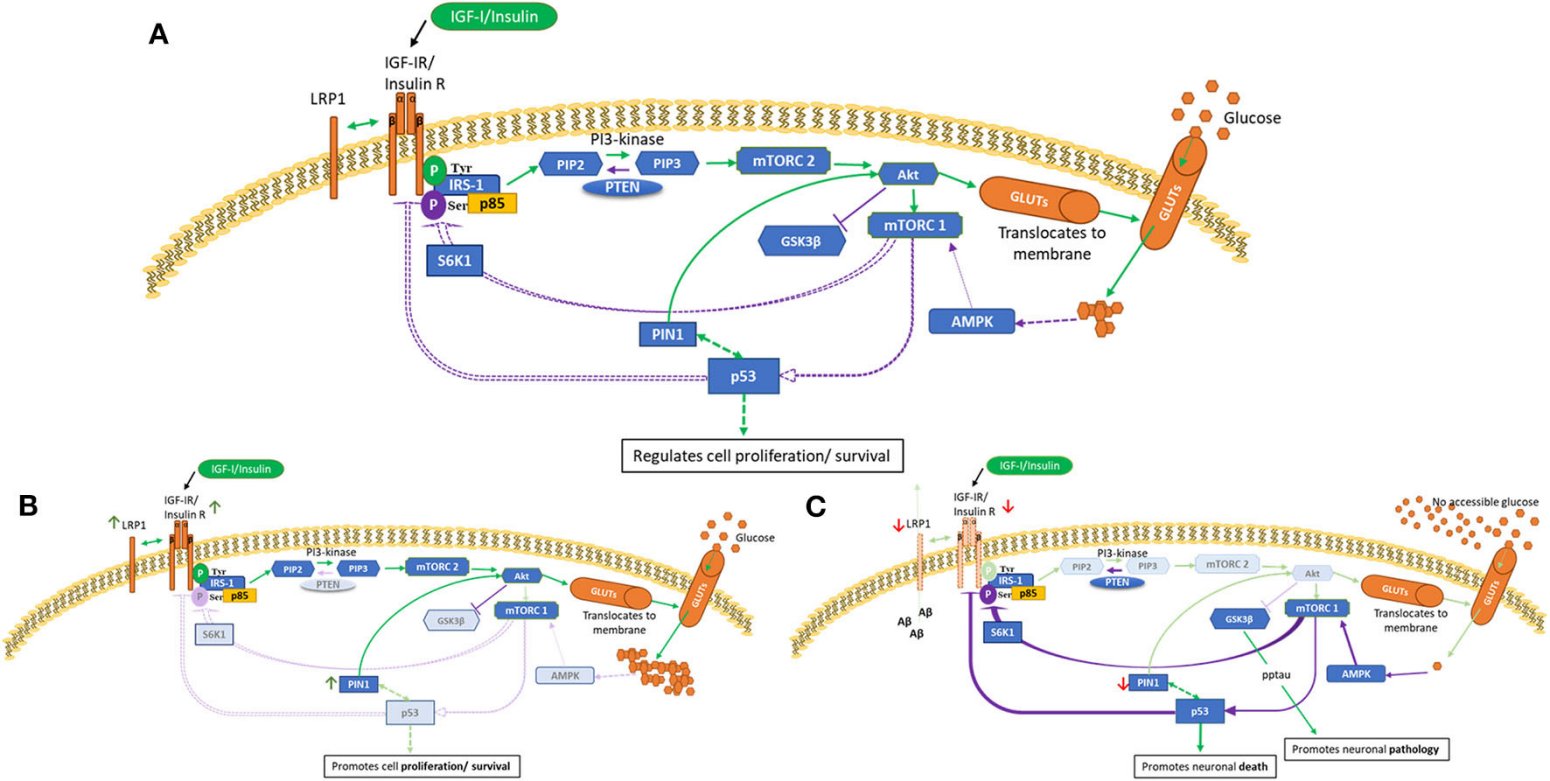
PI3K/Akt pathway in health **(A)**, cancer **(B)** and AD brain **(C)** cells. This is a schematic of the PI3K/Akt cellular pathway which regulates cell proliferation, metabolism and death. These figures attempt to highlight possible differences in cancer and AD compared with health. These indicated differences, as described in human and animal tissues and in cell culture, are meant to represent general concepts not specific cases. **(A)** shows normal regulation **(B)** indicates a cancer phenotype **(C)** illustrates AD as an insulin-resistant state i.e., T3DM. Green lines represent activation and purple lines represent feedback from the activation pathway. Activation of the IGF-1/insulin receptors leads to tyrosine phosphorylation of IRS-1 and activation of mTORC2 and Akt, resulting in glucose uptake. Homeostasis is maintained partly by mTORC1 sensing of metabolic conditions, which, as appropriate, leads to phosphorylation of p53 and S6K1 serine phosphorylation of IRS-1. p53 is a negative regulator of IGF/insulin receptors, IGF-II and glucose transporters. [A] Normal cellular homeostasis as described above [B] In cancer, negative feedback pathways are switched off leading to upregulation of proliferation, metabolism and cell survival. A modified genetic landscape (e.g., p53, PTEN) enables tumor cells to benefit from a glucose-rich, IGF/insulin-rich environment (insulin-resistance such as in T2DM).In cancer, Akt can phosphorylate and inactivate GSK-3β, which results in increased protein synthesis that supports cell growth. [C] In AD brain with insulin-resistance, or if, due to decreased blood flow there is no glucose accessible, the PI3K/Akt pathway is effectively switched off or downregulated. This leads to upregulation of GSK-β that culminates in tau phosphorylation and aggregation and increased amyloid beta production. Lack of intraneuronal glucose would trigger AMPK to activate mTORC1, p53, S6K1 serine phosphorylation of IRS-1. This could be a self-perpetuating cycle.

The phosphoinositide-3-kinase-(PI3K/Akt) signaling pathway, as depicted in Figure 1A, has been evolutionarily conserved to regulate and maintain appropriate cell growth, survival and metabolism. This schematic presents an overview of glucose utilization management within normal cells. Two major activators of this pathway are insulin and IGFs (31) which act via specific receptor tyrosine kinases, IGF-IR and the insulin (IR) receptors. The IR can be spliced to produce two isoforms, IR-A and IR-B. Upon ligand binding, the receptors can dimerize forming IR/IGF-IR hybrids which have different biological consequences depending upon the IR isoform present (32, 33). Generally, insulin acts via the IR, and IGF-I and IGF-II act via the IGF-IR and hybrid receptors. IR-A binds IGF-II and insulin, whereas IR-B has a higher affinity for insulin (34, 35). Emerging data have expanded our understanding of the complexity of these receptors and how they signal, in terms of their localization, trafficking and their ability to interact with other molecules (36). To ensure adequate fuel, insulin/IGF-I bind and activate IR/IGF-IR, causing tyrosine phosphorylation of insulin receptor substrate-1 (IRS-1), leading to Akt activation. This results in translocation of glucose transporter isoforms (GLUTs) (37) to the cell membrane enabling glucose uptake. Phosphorylation of mTORC1 initiates subsequent negative feedback mechanisms, such as serine/threonine phosphorylation of IRS-1, which are lost in a cancer phenotype (Figure 1B). mTORC1 (as opposed to mTORC2) is also considered a main regulator of autophagy, that maintains tissue homeostasis by degrading “abnormal” cellular contents (38). Aberrant autophagy occurs in and contributes to both cancer and AD, however, the impact of this is dependent on the stage of disease for both pathologies (39, 40).

Epidemiologic studies have shown that “higher” normal levels of circulating IGF-I are associated with a 25% increased risk of breast cancer, compared with “lower” normal levels (41). Overexpression of the IGF ligands and their receptors, IGF-IR, IR (particularly IR-A) and IGF-IR/IR hybrid receptors leads to increased activity of the PI3K/Akt pathway (36, 42–44). The IGF-IIR is a single, non-signaling, transmembrane receptor, enabling homeostasis by clearing excess IGF-II (45); thus loss of function mutations in the IGF-II receptor (46, 47) and/or loss of IGF-II gene imprinting (48) can lead to excess IGF-II available to activate the PI3K/Akt pathway. IGFBPs are often deregulated in cancer; IGFBP-2, for example, is often upregulated which intrinsically downregulates phosphatase and tensin homolog (PTEN) (49, 50) removing the inhibitory brake on the PI3K/Akt pathway. The cells compensate by upregulating glucose transporters, notably GLUT1, which substantially increases glucose importation into the cytoplasm (51, 52) and the cells switch to lactate fermentation (Warburg effect).

AD as an insulin-resistant state, by contrast is exemplified in Figure 1C. The brain has a high energy dependence, using about 20% of the body's resting energy requirement (~60% of glucose use) (53). Insulin crosses the BBB using a saturable transporter. Although GLUT1 and GLUT3 glucose transporters in the brain are insulin independent, the insulin dependent GLUT4 and GLUT8 are present in regions particularly affected in AD (54–56). IR (particularly IR-A) and IGF receptors are also strongly expressed in brain areas, such as the hippocampus, olfactory bulb, hypothalamus and cerebral cortex in neurones and glia and are important in memory formation in the hippocampus (55, 57, 58). Brain insulin and IGF levels are reduced in the aged brain with decreased insulin signaling and receptor activity (19, 59, 60), coinciding with decline in cognitive abilities. An early reduction of glucose uptake/metabolism is seen in pathology-related brain areas in AD and preclinical, pre-symptomatic subjects (61–63). Brain insulin-resistance is associated with impaired cognitive function (54) and is an important feature of AD in patients and in post-mortem tissue (64–69). Reduced insulin or IGF signaling leads to deficient uptake of glucose into neurones in those with mild cognitive impairment (MCI) who subsequently convert to AD, as well as being a major contributor to neuronal dysfunction and death in AD (70, 71). Reduced levels of insulin, IGF-I, II and their receptors associate with severity of pathology (19, 72). Furthermore, binding ability of these proteins is decreased, relative to increasing pathology (59, 73). In experimental studies, reduced IGF-I signaling was linked to increased deposition of Aβ (74, 75), phosphorylation of tau (76, 77), increased oxidative stress, neuro-inflammation and neuronal death (78). Of interest also, is the finding that the (non-toxic) monomeric form of Aβ can activate insulin/IGF-1 receptor signaling, and since these monomers aggregate in early AD, it is suggested that this may form a prelude to the disease process (79). Notably, systemic administration of IGF-I was able to lower the toxicity of Aβ in normal mice (80) and restore cognitive function in AD mouse models (81).

There are studies which are not in line with the hypothesis that IGF-I downregulation in AD is causative in the disease process but rather may be protective. The mixed results may partly lie in the fact that total IGF-I poorly reflects its bioactivity as most circulating IGF-I is bound to IGFBPs and will therefore be biologically inactive (82). There are also several variables between studies, for instance age of onset, stage of disease progression, presence of diabetes, or IGF-I gene polymorphisms.

Therefore, overall, in cancer and AD, the control of these pathways is compromised, allowing feed-forward and feed-backward cycles which lead either to cell over proliferation/deregulation or conversely death.

## Comparing Regulatory Molecules and Their Role in AD and Cancer

The PI3K/Akt pathway is kept in equilibrium by key regulators, some of these are briefly discussed here in terms of their effects on glucose metabolism in cancer and AD and are depicted in Figures 1A–C.

### IRS-1

IRS-1 plays a critical regulatory role in transmitting signals from IGF-IR/IR receptors via the PI3K/AKT pathway. It is commonly overexpressed in cancer and this has been associated with poor outcome for breast cancer patients (83), particularly if the tumor is positive for the estrogen receptor (84). Tyrosine phosphorylation activates and serine/threonine phosphorylation inhibits IRS-1 activity. Ribosomal protein S6 kinase beta-1 (S6K1) is one kinase responsible for inhibitory phosphorylation of IRS-1(85) and this negative feedback inhibition is lost in many cancers, including breast cancer (86).

In AD, insulin and IGF signaling is adversely affected in important brain areas. Phosphorylation of IRS-1 at serine 616 (pS616) and p-serine 636/639 are early markers of brain insulin-resistance, commonly present in MCI and AD (67). Aβ oligomers are thought to initiate IGF-I resistance and IRS-1 inactivation and to be associated with increased oligomeric Aβ plaques and memory impairment. Neurones in the temporal cortex in AD have been reported to show reduced levels of active IRS-1 and−2, but increased inactivated IRS-1, particularly at p-serine 312 and 616, and this was associated with NFT (73). Apart from indicating insulin-resistance and decreased glucose uptake, it suggests a relationship between IRS-1, tau (NFT) and Aβ pathology.

### p53 Tumor Suppressor Gene

Wild-type p53 regulates many cell functions including cell cycle arrest, apoptosis and metabolism (87). P53 negatively regulates IGF-IR, IGF-II, GLUTs 1 and 4 and positively stimulates IGFBP-3 (pro-apoptotic factor) (88–91). In cancer, including breast cancer, p53 is often mutated, resulting in a loss of its tumor suppressor activity (92–94). This disrupts regulation of IGF-IR, IGF-II, GLUTs 1, 4, and IGFBP-3, leading to enhanced activation of the PI3K/Akt pathway and glucose uptake. Increased Aβ positively correlates with p53 levels (91, 92). AD brain levels of p53 are thus increased, which promotes tau hyperphosphorylation and ultimately neuronal death (90).

### Peptidyl-Prolyl Cis-Trans Isomerase NIMA-Interacting-1 (PIN1)

Pin1 is a peptidyl-prolyl cis–trans isomerase (PPIase) able to isomerise p-serine/p-threonine-proline sequences thus effecting conformational change which alters the activity of its target proteins (95). It is highly expressed in many cancers (96, 97) and facilitates activation of the PI3K/Akt pathway. One way it does this is by increasing Akt stability through serine 473 phosphorylation (98). In breast cancer, high levels of both Akt-p-S473 and PIN1 predict a poorer prognosis than either alone (99). PIN1 can also induce a conformational change to the tumor suppressor gene p53 (100) and its overexpression in the presence of p53 mutations are prognostic for poor clinical outcome in breast cancer (101). SUMO protease-1 (SENP1) binds to, and deSUMOylates PIN1, and its levels correlate with those of PIN1 in breast cancer (102, 103). PIN1 is inhibited by *BRCA-1*, the tumor suppressor gene (104) suggesting that PIN1 would play an important role in the development of tumors in which *BRCA1* is mutated. PIN1 also supports increased cell proliferation by promoting glycolysis in tumor cells. This is achieved by stimulation of pyruvate kinase translocation (that catalyses the rate-limiting step during glycolysis) to the nucleus (95, 105). As a consequence of these functions, PIN1 inhibitors have been developed and shown to slow the progression of cancer (96).

In brain, PIN1 is located in neuronal dendrites and postsynaptic densities and its activity and expression are reduced in MCI and AD (106, 107), likely to make neurons more vulnerable to Aβ and increasing synaptic degeneration (108). Notably, PIN1 enables tau dephosphorylation via protein phosphatase PP2A and co-localizes with hyperphosphorylated tau in AD brain (109).

### Low-Density Lipoprotein Receptor–Related Protein 1 (LRP1)

The LRP1 receptor is a multifunctional receptor involved in many cellular functions including endocytosis and cell signaling. Notable is its intrinsic link with energy homeostasis; through its binding to the IGF-IR (110) and the IR (111), LRP1 plays a central role in insulin/IGF signaling affecting cell proliferation, survival, glucose and lipoprotein metabolism (112, 113).

The role that LRP1 plays in cancer is dependent upon the type of tumor and the cellular environment. In breast cancer, early reports indicated that a low expression of LRP1 correlated with more aggressive tumors (114). More recent work, however, consistently indicates a role for LRP1 in supporting breast cancer cell invasion and metastasis (115, 116) by increasing expression of matrix metalloproteinases (MMPs), MMP-2, and 9 (117).

In the brain, LRP1 is important for cell survival, lipoprotein metabolism and synaptic plasticity, and is highly expressed in neurones. It binds leptin, enabling leptin receptor phosphorylation and Stat3 activation. Deletion of the *Lrp1* gene in the mouse hypothalamus results in increased body weight (obesity) (118); conditional *Lrp1* brain knock-out produces glucose intolerance (111). LRP1 interacts with the insulin receptor, regulating insulin signaling and glucose uptake, and influencing GLUT3 and−4 glucose transporter levels (111). Insulin resistance in peripheral tissues in rodents involves loss of GLUT4 function (119, 120). Centrally, in the rat hippocampus, GLUT4 is vital to memory acquisition, inhibition causing memory impairment (56). Amyloid requires constant clearance pathways, LRP1 is known for its function as a clearance receptor able to remove amyloid across the BBB (121), but also to endocytose Aβ for elimination by lysosomes. LRP1 expression is reduced with age in mouse (122) and human brain (123), and to a greater degree in AD (122, 123). Notably, hyperglycaemia and increased insulin resistance, as in type-2 diabetes mellitus (T2DM), suppress LRP1 expression and exacerbate AD pathology in mice (111). Reduced LRP1 levels are associated with increased neuronal death (124) signifying that LRP1 is required for the neuroprotective effects of insulin signaling (125).

## Summary

The PI3K/Akt pathway is central to the sensing of metabolic and nutritional changes in our environment and is clearly deregulated in both cancer and AD. Considering that most of the risk factors for both, such as obesity, T2DM and smoking are modifiable through lifestyle changes, an effective strategy could be a preventive approach; for instance re-establishing physiological glucose levels by diet. This minireview, however, attempts to briefly explore some of the underlying mechanisms to identify possible therapeutic targets for these conditions, already ongoing. By addressing the apparent inverse relationship between cancer and AD we hope to identify regulatory molecules in the PI3K/Akt pathway important in cell proliferation and glucose utilization. In cancer this leads to upregulation of glucose uptake and cell proliferation, which contrasts with AD where there is lack of glucose availability, increased pathology, and consequent neuronal death. For both breast cancer and AD there has been a drive for the identification of biomarkers for early detection, ultimately to improve long-term survival. Notably, pre-clinical studies have identified IRS-1, p53, PIN1 and LRP1 as individual potential therapeutic targets (126–133) for both disease states, and changes in these are in themselves putative biomarkers.

These may provide alternative targets for future trials, but the possibility of inverse effects of altering these proteins, as we outline here, suggests that a delicate balance is required within the PI3K/Akt pathway. It is notable therefore that Metformin, an antihyperglycemic agent for diabetes, appears to promise some beneficial therapeutic outcome in both cancer and AD (134, 135). In cancer the mechanism is likely to be via mTOR inhibition and activation of p53 (136); in T2DM and T3DM-AD, it is probably the reduction of insulin-resistance (137). Whilst it is challenging to develop specific drugs for the clinical setting, understanding the regulatory aspects of this pathway may enable a co-targeting approach to reduce non-specific toxicity and increase specificity, thus achieving a better outcome.

## Author Contributions

SA-B and CP proposed the concept for the review. CP, SA-B, KB, JH and RB contributed to writing the paper. KB designed the figures. All authors contributed to the article and approved the submitted version.

## Conflict of Interest

The authors declare that the research was conducted in the absence of any commercial or financial relationships that could be construed as a potential conflict of interest.
